# Fish oil rich diet in comparison to saturated fat rich diet offered protection against lipopolysaccharide-induced inflammation and insulin resistance in mice

**DOI:** 10.1186/1743-7075-8-16

**Published:** 2011-03-09

**Authors:** Matam Vijay-Kumar, Sally M Vanegas, Nilam Patel, Jesse D Aitken, Thomas R Ziegler, Vijay Ganji

**Affiliations:** 1 Pathology and Laboratory Medicine, Department of Medicine, Emory University School of Medicine, Atlanta, GA 30322, USA; 2 Division of Nutrition, School of Health Professions, College of Health and Human Sciences, Georgia State University, Atlanta, GA 30302, USA; 3 Division of Endocrinology, Metabolism and Lipids, Department of Medicine, Emory University School of Medicine, Atlanta, GA 30322, USA

## Abstract

**Background and Objective:**

Systemic chronic inflammation is linked to metabolic syndrome, type-2 diabetes, and heart disease. Lipopolysaccharide (LPS), a Gram negative microbial product, triggers inflammation through toll-like-receptor-4 (TLR-4) signaling. It has been reported that dietary fatty acids also modulate inflammation through TLR-4. We investigated whether fish oil (FO) rich diet in comparison to saturated fat (SF) rich diet would confer protection from pathologies induced by LPS.

**Methods:**

Twenty C57BL/6 mice were divided into two groups. One group received FO-diet and other received SF-diet *ad libitum *for 60 days. Diets were isocaloric containing 45% energy from fat. After 60-days of feeding, blood was collected after overnight fast. Mice were allowed to recover for 4-days, fasted for 5-hours, challenged with 100 ng/mL of LPS intraperitonially, and bled after 2-hours. After 7-days of recuperation, mice were challenged with 500 ng/mL of LPS intraperitonially and observed for physical health.

**Results:**

Food intake was similar in FO- and SF-fed mice. FO-fed mice compared to SF-fed mice had significantly less body weight gain (P = 0.005), epididymal fat weight (P = 0.005), fasting blood glucose (70.8 vs 83.3 ng/dL; P < 0.05), HOMA-IR (5.0 vs 13.6; P < 0.019), and serum cholesterol (167 vs 94 mg/dL; P < 0.05). When challenged with LPS, FO-fed mice had significantly lower serum IL-1β compared to SF-fed mice (2.0 vs 30.0 pg/mL; P < 0.001). After LPS-challenge, SF-fed mice had higher mortality, lost more body weight, and had greater decrease in blood glucose compared to FO-fed mice.

**Conclusion:**

Overall, FO-diet compared to SF-diet offered protection against deleterious effects of LPS in mice.

## Introduction

Although inflammation is part of the body's normal response to infection and injury, extreme or inappropriate inflammation is linked to the pathobiology of several diseases [1]. Systemic low-grade chronic inflammation plays a major role in the pathogenesis of insulin resistance, metabolic syndrome, obesity, type 2 diabetes mellitus (DM2), and cardiovascular disease (CVD) [2-5]. Consequently, persons with obesity, DM2, and CVD experience increased markers of inflammation compared to their counterparts [6].

Toll-like-receptors (TLR) play an important role in the regulation of innate immunity [7-9] by detecting most pathogens such as bacteria, fungi, and viruses [10]. Toll-like-receptor-4 (TLR-4) is a subclass of the TLR family involved in activation of the innate immune and inflammatory response in mammals [9-11] via ligation of lipopolysaccharide (LPS), a Gram-negative bacterial endotoxin found on the outer cell membrane of bacteria. Activation of TLR-4 leads to the induction of genes for many inflammatory cytokines [9,12-14]. These cytokines are involved in inducing insulin resistance, glucose intolerance, and infiltration of macrophages into the adipose tissue which further leads to increased production of inflammatory markers [15-17]

Increased intake of saturated fat (SF) is linked to dyslipidemia and adiposity, which are further related to increased risk for DM2 and CVD [18]; while n-3 fatty acids present in fish oil (FO) are linked to decreased risk for these diseases [19]. However, the exact molecular mechanism linking dietary fatty acids with obesity, DM2, and CVD is not well understood. Recently, *in vitro *experiments have shown that TLR-4 responds to nonbacterial ligands such as lauric acid (a saturated fatty acid) and n-3 fatty acids such as eicosapentaenoic acid (EPA) and docosahexaenoic acid (DHA) [20-22]. Further, it has been shown that TLR-4 deficiency protects mice from inflammation, insulin resistance, and increased adiposity in mice fed a diet high in SF [23]. It is likely that TLR-4 is a possible link between dietary fatty acids and inflammation, insulin resistance, and DM2. *In vivo *studies are limited linking dietary fatty acids with TLR-4 signaling and inflammation, insulin resistance, and adiposity. Also, it is not known if FO rich diet offers protection against the TLR-4 agonist, LPS *in vivo*. Greater understanding of the role of TLR-4 in relation to dietary fat is important in understanding the pathobiology of insulin resistance and DM2. Therefore, the objective of this study was to investigate whether a diet rich in FO of marine origin in relation to a diet rich in SF would offer protection against endotoxemia (LPS)-induced pathologies in C57BL/6 mice.

## Methods

### Animals and facilities

Approval for the use of mice was obtained from the Institutional Animal Care and Use Committees of Emory University and Georgia State University, Atlanta, GA. Twenty, 4-week old male C57BL/6 mice were purchased from Jackson Laboratories (Bar Harbor, Maine, ME). All mice were housed in metal barred cages with bedding (5 mice/cage). Sufficient amounts of bedding were used to keep animals clean and dry between cage changes and to prevent odors. Animals had 12 hour light and dark cycles. Bedding changes, along with cleaning and sanitation of cages and associated equipment, such as watering devices, were performed as often as necessary.

### Diets and treatments

After arrival, mice went through a 4-day acclimatization period. During this period, mice were fed a regular chow diet. Twenty mice were randomly divided into two groups of 10 mice each. Diets rich in FO (product # D08092503) and SF (product # D05122102P) were purchased from Research Diets, Inc. (New Brunswick, NJ). Type of fat in FO and SF diets were menhaden oil and lard, respectively. The diets were isocaloric and contained 45% energy from total fat (Table 1**)**. Enough food was provided for mice to eat *ad libitum *for 2 days. After this time, any unconsumed food was thrown away after weighing and the amount of food eaten were quantified.

**Table 1:** Composition of experimental diets

Ingredient	Fish oil-based diet^1^		Saturated fat-based diet^2^	
	*g (%)*	*Kcal*	*g (%)*	*Kcal (%)*
Protein	23.7	20	23.7	20
Carbohydrate	41.4	35	41.4	35
Fat^1^	23.6	45	23.6	45
Casein	200 (23.3)	800	200 (23.3)	800
L-Cystine	3 (0.35)	12	3 (0.35)	12
Corn starch	72.8 (9.88)	291	72.8 (9.88)	291
Maltodextrin	100 (11.65)	400	100 (11.65)	400
Sucrose	172.8 (20.14)	691	172.8 (20.14)	691
Cellulose	50 (5.83)	0	50 (5.83)	0
Soybean Oil	25 (2.91)	225	25 (2.91)	225
Lard	0	0	177.5 (20.68)	1598
Menhaden oil	177.5 (20.68)	1598	0	0
Mineral mix	10 (1.17)	0	10 (1.17)	0
Vitamin mix	10 (1.17)	40	10 (1.17)	40
Choline bitartrate	2 (0.23)	0	2 (0.23)	0
Cholesterol	0.17 (0.02)	0	0.71 (0.08)	0
Total	858.2	4057	858.2	4057

^1 ^Source fish oil was menhaden oil

^2 ^Source saturated fat was lard

### Study design

Mice received FO and SF diets for 60 days. At the end of 60-days, all mice were fasted overnight and blood was collected using the retro-orbital method while mice were under isoflurane anesthesia. Following a 4-day recovery, both groups were fasted for 5-hours, challenged with 100 ng of LPS intraperitonially, and bled 2-hours post LPS-challenge. Then mice were allowed to recover for 7 days from the 100 ng LPS challenge. Following the recovery, mice were fasted for 5-hours, challenged with 500 ng of LPS intraperitoneally, After 2 hours, mice were then euthanized using carbon dioxide and epididymal fat, liver, colon, and cecum were harvested. Following 100 ng and 500 ng LPS challenges, mice were observed for any physical abnormalities including for mortality. During recovery phases, mice were fed their respective diets. Body weights were recorded on a weekly basis, at the end of 60-day period, and at the end of LPS challenge.

### Reagents and biochemical analysis

Hemolysis-free serum was generated by centrifugation of blood using serum separator tubes (Becton Dickinson, Franklin Lakes, NJ). Serum was stored at -80°C until analysis. Blood samples were analyzed using the HESKA CBC-Diff Veterinary Hematology System (Heska, Loveland, CO). Concentrations of IL-1β, IL-6, TNF-α, keratinocyte chemoattractant (KC), fasting blood glucose, serum insulin, and serum leptin were measured at the end of 60-days and after 100 ng of LPS challenge. IL-1β, IL-6, TNF-α, and KC were all analyzed using ELISA kits following manufacturer instructions. ELISA kits for serum IL-1β (catalog # DY401), IL-6 (catalog # DY406), TNF-α (catalog # DY410), and KC (catalog # DY453) were purchased from R&D Systems, Minneapolis, MN. Fasting blood glucose was analyzed with Accu-Check Advantage blood glucose meter (Roche, Florence, SC) by using blood from the tail vein. Serum insulin and leptin concentrations were analyzed using ELISA kits purchased from Linco Research Inc (St. Charles, MO). Escherichia coli LPS 0111:B6 was purchased from Sigma-Aldrich (St. Louis, MO). Homeostatic Model Assessment-Insulin Resistance (HOMA-IR) was calculated from insulin and glucose concentrations (fasting insulin (μU/mL) × fasting glucose (mg/dL)/405).

### Data analysis

The statistical analysis was performed using GraphPad Prism Software (Version 5.0, La Jolla, CA). Normality tests were done, after which parametric (unpaired t-test) or nonparametric (Mann-Whitney U) tests were used to analyze the differences between diets. The difference between pre- and post-LPS-treatment values within the diet group was analyzed with paired t-test for normal data or with Wilcoxin Sign test for nonnormal data. Mortality rate between SF and FO diets after the 100 ng and 500 ng LPS-challenge was analyzed with Fisher's exact test. Statistical significance was set at *P *≤ 0.05.

## Results

### Effects of FO and SF diets, after LPS-challenge on food intake and body and tissue weights

**Figure 1:**
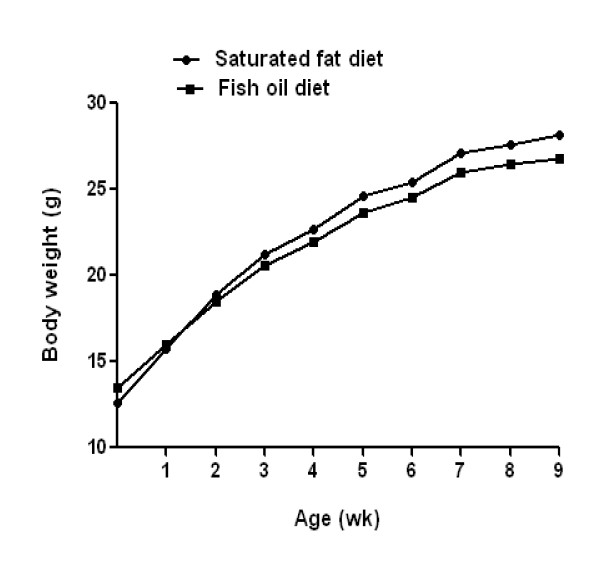
**Effects of fish oil (FO) and saturated fat (SF) diets on weight gain in C57BL/6 mice by week. Data points represent mean (*n *= 10)**. At the end of the 60 day feeding period, the end mean weights were not significantly different between SF and FO diet groups (28.2 vs. 26.8 g; *P *< 0.086). Significant differences were tested with Man-Whitney U test.

Total food intake during the 60-day period was not significantly different between mice that received FO and SF diet (*P *= 0.60). Average body weight changes for FO and SF diet groups are presented in Figure 1. Although there was no significant difference in body weights between SF and FO diet groups at the end 60 day period (28.2 vs. 26.8 g; *P *< 0.186), the net weight gain was significantly higher with SF diet compared to FO diet (15.6 g vs. 13.3 g; *P *= 0.005) (Table 2). Epididymal fat was significantly increased in mice that received SF diet compared to mice that received FO diet (0.61 g vs. 0.45 g) (*P *= 0.005). Of organ weights at necropsy, colon weight were significantly lower in SF diet group compared to FO diet group (*P *< 0.013) Cecum and liver weights were not significantly different between two diet groups (Table 2).

**Table 2:** The effect of fish oil and saturated fat on body and tissue weights^1^

	**Fish oil diet**	**Saturated fat diet**	**P-value**
	
End point body weight,^2 ^*g*	26.8 ± 0.37	28.2 ± 0.64	0.19
Net weight gain,^3 ^*g*	13.3 ± 0.47	15.6 ± 0.53^4^	0.005
Tissue weight^5^			
Epididymal fat, *g*	0.45 ± 0.02	0.61 ± 0.74^4^	0.005
Colon, *g*	0.15 ± <0.01	0.12 ± 0.01^4^	< 0.013
Cecum, *g* Liver, g	0.19 ± 0.01 1.01 ± 0.03	0.23 ± 0.02 0.99 ± 0.05	0.26 0.1

^1^Data are presented as mean ± SE

^2^Measured after 60 day feeding period

^3 ^Difference between 60 day feeding period weights and pre-study values

^4^Significantly different form saturated fat diet

^5^Weights, after 2 hours post 500 ng LPS-challenge

### Effect of FO and SF diets, after LPS-challenge on overall well-being and mortality of mice

**Figure 2:**
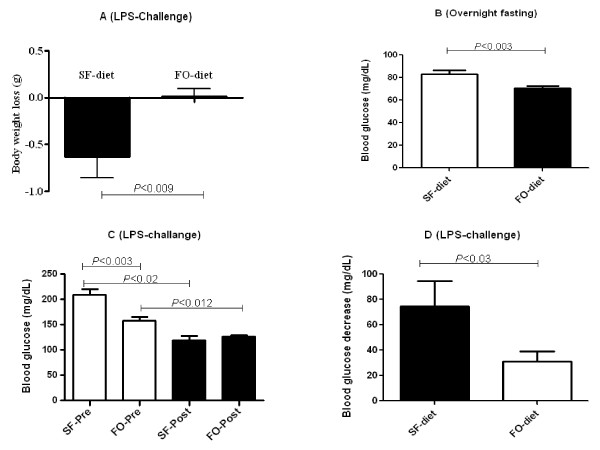
**Effect of fish oil (FO)and saturated fat (SF) diets on lipopolysaccharide (LPS)-induced body weight (A), overnight fasting (B), plasma glucose (C), and blood glucose drop (D)**. Body weight loss (A) calculation was based on weight changes between body weight at the end of 60 day period and body weight at the end of 7 day recovery post 100 ng LPS challenge (*n *= 6). Overnight fasting (12 h) blood glucose concentrations of mice fed FO and SF diets at the end of 60 day are shown in B (*n *= 10). Blood glucose (C) and blood glucose drop (D) for mice that received FO and SF diets before and after LPS-challenge (*n *= 5-9). Blood glucose drop was calculated based on blood glucose between pre- and post-100 ng LPS-challenge (*n *= 5). Significant differences were tested either with unpaired/paired t-test or with Man-Whitney U test/Wicoxin Sign test. Values are means ± SE. Only significant comparisons are presented.

Mice that received SF diet in comparison to FO diet were moribund. After LPS-challenge, mice that received SF diet exhibited less or no nesting behavior. After 100 ng LPS challenge, 1 mouse died in SF diet group but none in the FO diet group. After 500 ng LPS challenge, 1 mouse died in FO diet group and 3 mice died in SF diet group. However, the mortality rate between SF and FO diets after 500 ng LPS-challenge was not significant in Fisher's exact test. After LPS challenge, mice in SF diet group compared to FO diet group had significantly more weight loss (-0.63 g vs. -0.22 g; *P *< 0.009) (Figure 2A).

### Effect of FO and SF diets, after LPS-challenge on glucose, insulin, and HOMA-IR

To investigate the effect of TLR-4 signaling on glucose metabolism, we injected LPS, the classic TLR-4 agonist, intraperitonially at a low dose of 100 ng/mouse. To investigate the impact of FO diet in comparison with SF diet on glucose metabolism, we tested blood glucose concentrations after a 12-hour overnight fast at the end of 60 day feeding. FO diet group had significantly lower overnight fasting blood glucose concentrations compared to SF diet group (70.8 mg/dL vs 83.3 mg/dL) (*P *< 0.014) (Figure 2B). Both groups of mice showed a significant drop in blood glucose concentrations after LPS-challenge. However, no significant difference was observed in blood glucose between FO- and SF-fed mice after LPS- challenge (Figure 2C). A greater decrease in blood glucose concentration was observed with SF diet compared to FO diet after 100 ng of LPS challenge (89.2 mg/dL vs 30.9 mg/dL; *P *< 0.03) (Figure 2D).

**Figure 3:**
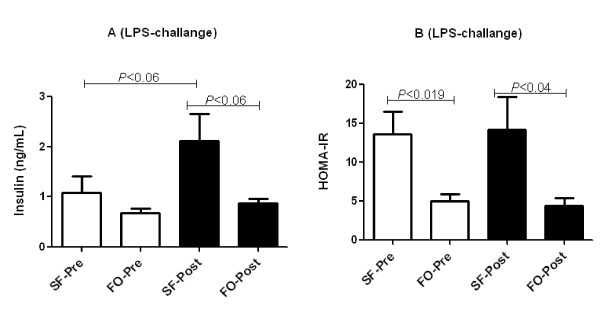
**Effect of fish oil (FO) and saturated fat (SF) diets on serum insulin (A) and Homeostatic Model Assessment-Insulin Resistance (HOMA-IR) (B) before and after 100 ng lipopolysaccharide (LPS)-challenge in C57BL/6 mice (*n *= 5-10)**. Serum insulin (A) and HOMA-IR (B) for mice that received FO and SF diets before and after LPS-challenge (*n *= 5-9). Significant differences were tested either with unpaired/paired t-test or with Man-Whitney U test/Wicoxin Sign test. Values are means ± SE. Only significant comparisons are presented.

After administration of 100 ng of LPS, SF-fed mice had a higher mean insulin concentration compared to FO-fed mice (2.11 vs. 0.88 ng/mL) (Figure 3A). However, these values were not significant (*P *< 0.06). We calculated HOMA-IR scores from fasting blood glucose and insulin concentrations to assess whether FO diet protected mice from IR. In fact, mice that received FO diet had significantly lower HOMA-IR scores compared to mice that received SF diet after 60-day feeding (5.0 vs. 13.6; *P *< 0.019) and after LPS-challenge (4.43 vs. 14.23; *P *< 0.04) (Figure 3B).

### Effect of FO and SF diets, after LPS-challenge on serum cholesterol, leptin, and cytokines

**Figure 4:**
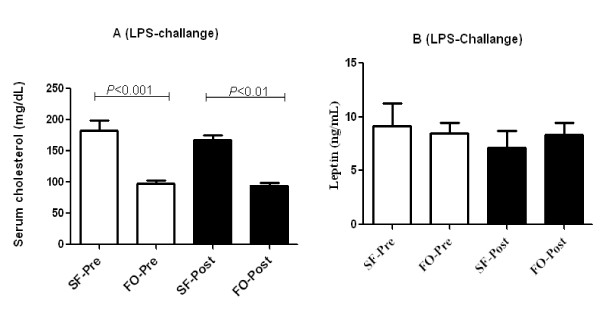
**Effect of fish oil and saturated fat diets on concentrations of serum cholesterol (A) and leptin (B) before and after 100 ng lipopolysaccharide-challenge in C57BL/6 mice (*n *= 5-10)**. Values are means ± SE. Significant differences were tested either with unpaired/paired t-test or with Man-Whitney U test/Wicoxin Sign test. Only significant comparisons are presented.

After 60 days of feeding, FO diet group had significantly lower serum cholesterol concentrations when compared to SF diet group (97.6 mg/dL vs 183 mg/dL) (*P *< 0.01). As expected, SF diet was hypercholesterolemic compared to FO diet after 60 days of feeding (*P *< 0.001). These differences persisted between SF and FO diets even after LPS challenge. LPS administration had no impact on serum cholesterol (Figure 4A). There was no difference in mean serum leptin between the FO and SF diet groups at baseline or after LPS-challenge (Figure 4B).

**Figure 5:**
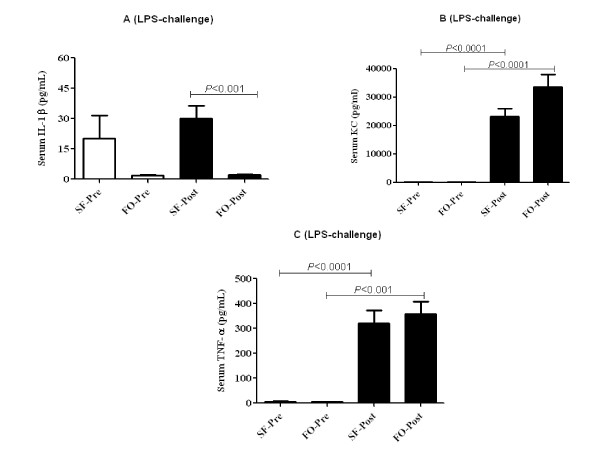
**Effect of fish oil and saturated fat diets on concentrations of serum IL-1β (A), KC (B), and TNF-α (C) before and after 100 ng lipopolysaccharide-challenge in C57BL/6 mice (*n *= 5-10)**. Values are means ± SE. Significant differences were tested either with unpaired/paired t-test or with Man-Whitney U test/Wicoxin Sign test. Only significant comparisons are presented.

Mice that received SF diet had significantly higher mean serum IL-1β concentrations compared to those that received FO diet after 100 ng LPS-challenge (30.0 pg/mL vs. 2.0 pg/mL) (*P *= 0.0004) (Figure 5A). As expected, with LPS-challenge, both in FO- and SF-fed mice had significantly higher concentrations of inflammatory markers such as serum TNF-α and KC. However, mean serum concentrations of KC (Figure 5B), TNF-α (Figure 5C), and IL-6 (data not shown) were not significantly different between mice that received FO and SF diets either at pre-LPS-challenge or post- LPS challenge.

## Discussion

To our knowledge, this is the first study that reported the protective effect of a diet rich in FO in relation to a diet rich in SF against LPS-induced pathologies in mice with functional TLR-4. SF-fed mice that were challenged with LPS got sicker and had higher mortality rate compared to FO-fed mice. After LPS challenge, SF-fed mice lost more weight and had greater drop in blood glucose concentrations compared to FO-fed mice. These coincided with higher serum IL-1β concentrations in SF-fed mice challenged with LPS compared to their counterparts. Additionally, net body weight gain, abdominal fat weight, serum cholesterol, and blood glucose were significantly higher in SF-fed mice compared to FO-fed mice.

Although the food intake during the 60 day feeding was not significantly different between FO- and SF-fed mice, the net body weight gain and epididymal fat weight were significantly increased in SF-fed mice suggesting that the weight gain observed in SF-fed mice was due to type of fat administered and not due to hyperphagia. Generally, increased adiposity is a cause for elevated inflammation and further fat intake/infusion is likely to lead elevated free fatty acids which further leads to elevated inflammation [24-26]. Intake of n-3 fatty acids from marine sources reduces adiposity by inhibiting the sterol regulatory element binding protein-1 and thereby decreasing the expression of lipogenic genes (acetyl CoA carboxylase and fatty acid synthase), increasing the gene expression of lipolytic genes (hormone sensitive lipase and serum amyloid A) [27], and decreasing expression of perilipin, a lipid droplet-protective protein [28]. Similar to our findings, Samane et al [29] found that FO decreased visceral adiposity compared to a diet rich in lard. They also reported no difference in weight gain between mice fed a high fat diet with 6% of fat replaced by fish oil or argan oil (mainly oleic acid) for 4 weeks, suggesting the effect of fish oil on body weight is affected by the duration and amount of fish oil intake.

Another beneficial effect of FO diet in relation to SF diet is decreased circulating cholesterol. After 60-day feeding, on average, mice fed FO diet had 87.5% lower serum cholesterol compared to mice fed SF diet. Depressed circulating cholesterol is a well established cardioprotective factor [30]. Generally, decreased blood cholesterol is due to lowered LDL cholesterol. It has been well documented diets rich in FO are cardioprotective [31,32]. However, effects of FO on blood lipids have not been uniform [30]. Chang et al [32] reported that the cardioprotective effect of FO in comparison to SF diet was not related to blood cholesterol. In their study, SF and FO diets had similar increase in blood cholesterol but free fatty acids and triglycerdes were lower with FO diet in relation to SF diet. Increased blood cholesterol was likely due to higher cholesterol content of SF and FO diets [32]. Additionally, cardioprotective effect of n-3 fatty acids compared to SF was due to reduced total LDL uptake and cholesterol ester deposition in arterial walls [32,33].

Studies have shown that diets high in fat (other than FO) and specifically high in SF induced metabolic endotoxemia and adipose tissue inflammation by altering gut microbiota and permeability to LPS [34-36] and by stimulating TLR-4 expression [37]. Antibiotic treatment reversed these effects significantly [34]. Thus metabolic endotoxemia (increased circulating LPS) may partly explain the increased inflammation with SF diet. In our study, the SF diet group had a significant decrease in colon weight compared to FO diet group (P < 0.013). This decrease in colon weight observed in the SF group may be a result of atrophy due to a change in the intestinal microbiota or increased colon weight in FO diet group due to protective effect of FO on the gut. More research investigating the effect of saturated fat and n-3 fatty acids on intestinal microbiota and pathobiology of gut is needed to verify this observation.

After administration of LPS, mice were observed for changes in behavior. In the post-500 ng LPS phase, 3 mice died in the SF group out of 9 (33.3% mortality rate) and 1 mouse died out of 10 (10% mortality rate) in the FO group suggesting that FO offered protection against LPS-induced sickness and mortality. Furthermore, after LPS-challenge, SF-fed mice had lost more body weight and experienced a greater decline in blood glucose (decrease was 42.8% with SF and 19.7% with FO) than FO-fed mice suggesting that the SF-fed mice were in a more catabolic state. Following the 60 day period, we noticed no overt in-between group differences in the physical appearance of mice. However, following 100 ng LPS-challenge, the SF diet group mice became reclusive and shortly fell asleep. In contrast, the FO-fed mice exhibited no abnormal behavior. After the 500 ng LPS-challenge, both groups of mice were moribund. However, mice in FO diet group recovered quicker (within 30 minutes) than the mice in SF diet group. These observations illustrate an over-all protective effect of fish oil from LPS-induced ill health.

After 60 days of feeding, FO diet group had a 17.7% decrease in fasting blood glucose compared to SF diet group (*P *< 0.01) suggesting overall protection of FO from hyperglycemia. Flachs P et al [38] reported a ≈4% decrease in blood glucose in mice fed a FO diet. Rossmeisl et al [39] reported a blood glucose decrease of ≈19.2% in mice fed DHA diet compared to mice fed a corn oil diet for 4 months. In contrast, Samane et al [29] reported no significant decrease in fasting blood glucose. It is likely that the differences in results in between these studies may be due to differences in duration of feeding and amount of FO present in experimental diets.

Although insulin concentration did not reach statistical significance, there was higher insulin concentration in SF group compared to FO group after mice were challenged with LPS (*P *< 0.06). FO diet group compared to SF diet group had lower HOMA-IR values after 60 day feeding and after LPS challenge. The observation of improved insulin sensitivity in rodents fed FO diet compared to rodents fed SF diet has been previously reported [39]. Differential effects of saturated and n-3 fatty acids on TLR-4 signaling may explain the variation in insulin resistance. Saturated fatty acids and LPS induce dimerization and recruitment of TLR-4 to the membrane which are essential for TLR-4 signaling. In contrast, DHA of FO inhibits the dimerization and recruitment of TLR-4 into the membrane [40] leading to attenuated TLR-4 signaling. *In vivo *studies have reported that FO is incorporated into the plasma membrane where it inhibits downstream signaling of TLR-4 [41,42] leading to decreased insulin resistance [43-45].

Obesity and DM2 are characterized by elevated circulating concentrations of inflammatory markers [46]. These inflammatory markers originate from adipose tissue [47]. In diet-induced obesity, macrophages infiltrate into white adipose tissue and these infiltrated macrophages produce inflammatory markers such as IL-1β [48,49]. We found a protective effect of FO against IL-1β, a potent inflammatory marker, in LPS-challenged mice. Several studies have demonstrated that consumption of FO decreased production of IL-1β from mononuclear cells [50-52]. Eevated IL-1β promotes insulin resistance [53]. IL-1β is produced from large pro-inflammatory protein complexes called inflammasomes [54]. These are part of innate immune system activated by various microbial stimuli. Other mechanisms through which FO exerts anti-inflammatory properties include displacement of arachidonic acid in the plasma membrane leading to the synthesis of anti-inflammatory PGE3 and LTB5 instead of PGE2 and LTB4, respectively [55]. Recent studies revealed that n-3 fatty acids produce E- and D-series resolvins [56-58] which are found to be potently anti-inflammatory [59].

There is a possibility that the overall beneficial effect of FO against LPS-induced ill health that we found in our study may also be due to mechanisms unrelated to TLR-4 and NRLs. Although n-3 fatty acids from FO are known to antagonize the effects of LPS-induced TLR-4 signaling, the protection of FO diet could potentially be due to increased production of anti-inflammatory components of n-3 fatty acid-derived eicosanoids. Based on the findings from this study, consumption of a diet rich in n-3 fatty acids from marine sources is beneficial against the deleterious effects of Gram negative bacterial infection.

## Competing interests

M. Vijay-Kumar, S. Vanegas, N. Patel, J. D. Aitken, T. R. Ziegler, and V. Ganji have no conflict of interest.

## Authors' contributions

MVK, TRZ, and VG designed research and conducted research; MVK, SMV, NP, and JDA conducted animal study, collected data, and analyzed data; VG was the principal investigator and obtained funding for the research, supervised the study, wrote the manuscript, and revised the manuscript. All authors read and approved the final manuscript.
